# Exploring P2X7 receptor antagonism as a therapeutic target for neuroprotection in an hiPSC motor neuron model

**DOI:** 10.1093/stcltm/szae074

**Published:** 2024-10-17

**Authors:** Alexandra E Johns, Arens Taga, Andriana Charalampopoulou, Sarah K Gross, Khalil Rust, Brett A McCray, Jeremy M Sullivan, Nicholas J Maragakis

**Affiliations:** Department of Neurology, The Johns Hopkins University School of Medicine, Baltimore, MD 21205, United States; Department of Neurology, The Johns Hopkins University School of Medicine, Baltimore, MD 21205, United States; Department of Neurology, The Johns Hopkins University School of Medicine, Baltimore, MD 21205, United States; Department of Neurology, The Johns Hopkins University School of Medicine, Baltimore, MD 21205, United States; Department of Neurology, The Johns Hopkins University School of Medicine, Baltimore, MD 21205, United States; Department of Neurology, The Johns Hopkins University School of Medicine, Baltimore, MD 21205, United States; Department of Neurology, The Johns Hopkins University School of Medicine, Baltimore, MD 21205, United States; Department of Neurology, The Johns Hopkins University School of Medicine, Baltimore, MD 21205, United States

**Keywords:** hiPSC, ALS, ATP, P2X7, purinergic signaling

## Abstract

ATP is present in negligible concentrations in the interstitium of healthy tissues but accumulates to significantly higher concentrations in an inflammatory microenvironment. ATP binds to 2 categories of purine receptors on the surface of cells, the ionotropic P2X receptors and metabotropic P2Y receptors. Included in the family of ionotropic purine receptors is P2X7 (P2X7R), a non-specific cation channel with unique functional and structural properties that suggest it has distinct roles in pathological conditions marked by increased extracellular ATP. The role of P2X7R has previously been explored in microglia and astrocytes within the context of neuroinflammation, however the presence of P2X7R on human motor neurons and its potential role in neurodegenerative diseases has not been the focus of the current literature. We leveraged the use of human iPSC-derived spinal motor neurons (hiPSC-MN) as well as human and rodent tissue to demonstrate the expression of P2X7R on motor neurons. We extend this observation to demonstrate that these receptors are functionally active on hiPSC-MN and that ATP can directly induce death via P2X7R activation in a dose dependent manner. Finally, using a highly specific P2X7R blocker, we demonstrate how modulation of P2X7R activation on motor neurons is neuroprotective and could provide a unique pharmacologic target for ATP-induced MN death that is distinct from the role of ATP as a modulator of neuroinflammation.

## Graphical Abstract

**Graphical Abstract:**
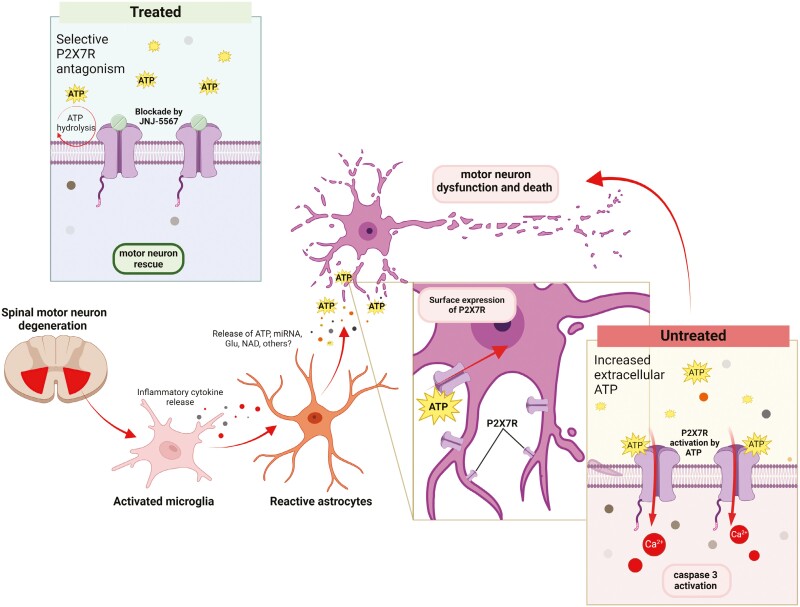

**Significance statement**

While the effects of increased extracellular ATP on non-neuronal cells involved in neuroinflammation have been well studied, its direct effects on human motor neurons remains unknown. Our results demonstrate that increased extracellular ATP is directly toxic to hiPSC-derived spinal motor neurons due to the activation of P2X7R on their surface. Furthermore, we demonstrate that the blood brain barrier-permeable P2X7R-specific antagonist JNJ-47965567, blocks the rise of intracellular calcium influx as well as caspase 3 activation in motor neurons subjected to increased extracellular ATP, providing proof of principal that P2X7R blockers could represent a novel therapeutic strategy in motor neuron disease.

## Introduction

Adenosine triphosphate (ATP) is a potent signaling molecule that has well characterized roles in inflammation as a damage associated molecular pattern (DAMP) that is released from cells in response to stress or damage and serves to stimulate and recruit immune cells to the site of injury.^1,2^ In the central nervous system, extracellular ATP participates in functions beyond those involved in initiating an immune response, including the regulation of homeostasis in neurons, astrocytes, and microglia,^3^ as well as the regulation of synaptic transmission and neuromodulation as both a neurotransmitter and a gliotransmitter that is co-released with glutamate and acetylcholine for intercellular communication.^4,5^

ATP acts by binding to purine receptors on the cell surface and triggers both ionotropic and metabotropic downstream signaling cascades. Included in the family of ionotropic purine receptors is P2X7R, a non-specific cation channel that has unique functional and structural properties compared to all other P2X receptors, making it a desirable pharmacological target for investigating the role of ATP in neuronal cell death. P2X7R has an activation threshold that is approximately 10 times greater than other ionotropic purine receptors, with an EC_50_ value for ATP of ~100 µM.^6^ During prolonged activation with ATP, P2X7R do not desensitize like other P2X receptors but rather trigger the formation of a non-specific pore in the membrane that allows molecules up to 900 Da to permeate, the role of which has yet to be made clear.^7,8^ The properties of the P2X7R that make it unique among other ATP-gated ionotropic receptors suggest that the P2X7R has distinct roles under pathological conditions of high extracellular ATP release.^1,9^

Despite the evidence from rodent models which suggests that functional P2X7R are present on embryonic-derived motor neurons,^10^ human studies have focused on characterizing the expression of P2X7R almost exclusively in astrocytes and microglia due to the receptor’s known involvement in inflammatory processes.^11-14^ Since the expression of P2X7R has not been examined on human induced pluripotent stem cell (hiPSC)-derived motor neurons (hiPSC-MN), we examined the expression of P2X7R in hiPSC-MN as well as in mouse and human spinal cord tissue. Furthermore, we show that these receptors are expressed at the membrane surface and are functionally active as evidenced by changes in calcium influx following activation with an ATP agonist, BzATP.

Given the importance of extracellular ATP for neuronal function, changes in purinergic signaling as a result of increased extracellular ATP has gained attention as a potential pathogenic mechanism underlying neurodegenerative diseases.^15^ The direct effect of extracellular ATP on human motor neuron survival has not previously been investigated and could represent a unique pathway that contributes to motor neuron death in motor neuron disease. Using a human iPSC platform, we propose that direct P2X7R activation on motor neurons is a relevant pharmacological target for neuroprotection in the context of increased extracellular ATP by demonstrating that the specific P2X7R antagonist, JNJ-47965567 (JNJ-5567), effectively rescues ATP-induced MN death. With this work, we propose that P2X7R is a potent mediator of motor neuron death and represents an important point of convergence between motor neuron survival and the neuroinflammatory axis.

## Materials and methods

### Human tissue

Human cervical spinal cord, occipital cortex, and motor cortex samples were obtained from the “Target ALS - Multicenter Human Postmortem Tissue Core” (http://www.targetals.org/human-postmortem-tissue-core/). The cervical spinal cord sample used in this study is from an 80-year-old female and represents a non-ALS control.

### Mouse tissue

Transgenic mice carrying the normal human SOD1 gene (B6SJL-Tg (SOD1) 2Gur/J, stock #002297) and the human SOD1 gene with the G93A mutation (B6SJL-Tg (SOD1*G93A) 1 Gur/J: Stock # 002726)^16,17^ were used for the study and are denoted as mSOD1. Endstage for mSOD1 mice was considered days 120-140, defined by phenotypic criteria, and was compared to B6SJL littermate control mice at the equivalent timepoint.

### Immunohistochemistry of mouse and human cervical spinal cords and human cortex

Frozen tissue sections were sectioned at 20 µm thickness and mounted on glass slides for storage at −80º prior to staining. Slides were left to rest at room temperature for 10 minutes before rinsing 3 times for 10 minutes in 1XTBS. The sections were then blocked with 10% donkey serum containing 0.3% triton-X for 1 hour. A primary antibody for SMI32 was added in 2% donkey serum and 0.3% triton overnight at 4 °C followed by a biotinylated anti-mouse IgG secondary antibody for 1 hour at room temperature. This process was repeated for all other relevant primary antibodies in 2% donkey serum and 0.2% triton overnight at 4 °C followed by incubation in the species-specific secondary antibodies for 2 hours at room temperature. All information about primary antibodies is listed in **Table 1**. The sections were then washed and mounted with Prolong gold with DAPI (Life Technologies) and stored until ready to image.

**Table 1.** List of primary antibodies and pharmacological compounds.

Antibody	Species	Staining Dilution	WB dilution	Company Cat. #
P2X7R intracellular epitope	Rabbit	1:100	1:100	Alomone Labs (#APR004)
P2X7R extracellular epitope	Rabbit	1:100	1:100	Alomone Labs (#APR008)
P2X4R	Rabbit	1:50	—	Abcam (#AB99541)
ARTC2	Mouse	—	1:100	Santa Cruz (#SC-515135)
Actin	Rabbit		1:5000	Cell Signaling (#4967)
GAPDH	Mouse		1:5000	Millipore Sigma (#G8795)
N-Cadherin	Mouse	—	1:5000	Cell Signaling (#14215)
ChAT	Goat	1:100	—	Millipore Sigma (#AB144P)
Isl1	Rabbit	1:100	—	Santa Cruz (#SC-30200) ThermoFisher (#PA5-27789)
Anti-beta III Tubulin	Mouse	1:200	—	Abcam (#AB78078)
Anti-beta III Tubulin	Rabbit	1:200		Abcam (#AB18207)
CTIP2	Rat	1:200	—	Abcam (#AB18465)
Caspase 3	Rabbit		1:1000	Cell Signaling (#9662)
SMI32	Mouse	1:2500	—	Biolegend (#801701)
Anti-mouse IgG, biotin	Mouse	1:5000	—	Invitrogen (#A16015)
GFAP	Chicken	1:200	—	Millipore Sigma (#AB5541)
CD68	Rat	1:200	—	Serotec (#MCA-1957)
Reagent	Working concentrations			Company Cat. #
BzATP	1 µM, 10 µM, 100 µM, 200 µM, 300 µM, 1 mM			Sigma Aldrich (#B6396)
JNJ-47965567	300 nM, 1 µM, 3 µM			Tocris (#5299)
NAD+	1 µM, 20 µM			Roche (NAD100-RO)

### RNA sequencing

All analyses were performed in R (v03.1.+446) using the Seurat package (v4.3.0). The Seurat objects used for these analyses were created by the original authors^18,19^ and were analyzed following the specifications described.

#### Human cervical spinal cord

RNA sequencing was conducted on cervical spinal cord tissue from a 56-year-old male donor who died from acute intracranial hemorrhage. Details about tissue preparation can be found in the original author’s manuscript.^19^ Briefly, feature counts were normalized following the removal of mitochondrial reads using the “LogNormalize” function. Principal component analysis was performed on 33 000 variable features followed by UMAP clustering using 50 components and a clustering resolution of 2.5. Fifty-three clusters of neurons were identified with this method of which Gautier et al, identified cluster 50 as the motor neuron cluster based on its enrichment for known cholinergic and motor neuron marker genes. To look at P2X7R expression specifically in MN, the data corresponding to cluster 50 underwent additional UMAP clustering using the top 2 components and a clustering resolution of 0.4. With these parameters 2 distinct clusters became apparent which authors identified as alpha and gamma MN subtypes based on the expression of marker genes that are present in mice and conserved in humans. Normalized expression data was used to generate all feature plots, ridge plots and violin plots.

#### Human lumbar spinal cord

The Seurat object for this analysis was received from the authors directly and consisted of 7 datasets generated from human lumbar spinal cord tissue from donors between the ages of 50 and 80 that were integrated using “SCTtransform” normalization. Principal component analysis was performed on the integrated dataset followed by UMAP clustering of major cell types using 30 components and a clustering resolution of 0.6. Cell types were manually annotated based on the enrichment of cell-type specific markers identified by the authors.^18^ For motor neuron-only visualization, clustered data were subset by “subtype annotation” such that individual feature plots could be generated for each cell type.

### Human spinal cord, oculomotor, and Onuf’s nucleus motor neuron analysis

Raw count data from laser captured motor neurons from human post-mortem tissue was generated as described in previous publications.^20,21^ Data were downloaded from the NCBI under the GEO Accession GSE93939 and analyzed using R. Expression data are presented as LogNormalized counts and compared between motor neuron types.

### Regional patterning protocol for the generation of human induced pluripotent stem cells (hiPSC)-derived neurons and astrocytes

Neural progenitors (NPC) were differentiated from human induced pluripotent stem cells (hiPSC) following a spinal cord, and for noted experiments, a cortical patterning protocol that we and others have described previously.^22–25^ In the spinal cord protocol, the neuroectoderm fate was induced through dual SMAD inhibition followed by the specification of caudal and ventral fates using retinoic acid and purmorphamine, respectively.^22,23^ In the cortical patterning protocol, hiPSC were initially lifted in suspension to generate three-dimensional embryoid bodies, then plated to generate neural rosettes, which cell-intrinsically (ie, in the absence of morphogens) develop a rostral and dorsal identity.^24,25^ The NPC generated after 30 and 22 days in vitro (DIV) for the spinal cord and cortical protocol, respectively, were then differentiated into motor neurons (MN) for additional 25-30 days in the presence of neuronal differentiating medium (NDM). To prevent the proliferation of astrocytes, neuronal cultures were treated once with 0.02 µM of cytosine arabinoside (Ara-C) (Millipore Sigma) for 48hrs. We and others have previously demonstrated that the spinal cord protocol generates highly pure MN cultures that express choline acetyl transferase (ChAT), a marker for mature MN^22,23^ and are relatively devoid of GFAP astrocytes. The cortical patterning protocol, generates forebrain neurons, including a population of COUP-TF-interacting protein 2 (CTIP2)-positive corticospinal motor neurons.^26^ To generate human induced pluripotent stem cell-derived astrocytes (hiPSC-A) expressing glial fibrillary acidic protein (GFAP), NPC were differentiated up to 90 DIV, in serum containing media and deprived of neuronal growth factors, to allow for the gliogenic switch to occur. For individual experiments, hiPSC-MN were plated onto 24 well plates or 6 well plates that had been previously coated in polyornithine (PLO) (100 µg/mL in PBS) and laminin (10 µg/mL in PBS) at a density of 50 000 or 350 000, respectively. Human iPSC-A were plated on matrigel coated 6-well plates at a density of 200 000 cells/well. All hiPSC lines used in this study represent control cell lines: CS8PAA, CS9XH7, CS0002, CIPS.

### Immunocytochemistry in hiPSC-MN

HiPSC-MN were passaged to 24 well plates that contained PLO-laminin coated 12 mm glass coverslips (ThermoFisher, 12CIR-1). HiPSC-MN were plated at a density of 50 000 cells/well and were cultured in NDM until DIV 60. HiPSC-MN were fixed with 4% PFA for 10 minutes followed by permeabilization with 0.1% Triton for 15 minutes. HiPSC-MN were not permeabilized prior to staining with the extracellular epitope directed P2X7R antibody and instead were treated with PBS for the equivalent amount of time. HiPSC-MN were then blocked with 3% BSA for 1 hour at RT followed by treatment with primary antibodies Tuj1, and either intracellular or extracellular epitope directed P2X7R, overnight at 4 °C. Cells were incubated for 2 hours at RT in the appropriate secondary antibodies raised in donkey before mounting with Prolong gold with DAPI (Life Technologies).

### Immunoblot (Western)

Cells were dislodged with 0.05% Trypsin followed by centrifugation at 300G for 5 minutes. Pelleted cells were resuspended vigorously in T-PER with phosphatase and protease inhibitors before being vortexed periodically, every 10 minutes. Samples were stored at −80 °C prior to running the Western blot. Samples were thawed on ice before 15 minutes of sonication followed by 20 minutes of centrifugation at 12 000 rpm. Samples were heated for 10 minutes at 70°C, and 15µg of total protein loaded into Bis-Tris gels for SDS-polyacrylamide gel electrophoresis. Samples were run for 2 hours in MES running buffer followed by rapid semi-dry transfer onto PVDF membranes. Membranes were blocked in 5% milk reconstituted from powder in TBS-T. All primary antibodies were incubated overnight at 4 °C followed by secondary staining with anti-rabbit or anti mouse hRP-conjugated antibodies in 5% milk. Immunoreactivity was visualized with enhanced chemiluminescence. Western blot on human and mouse tissue was performed similarly.

### Biotin-streptavidin pulldown

Human iPSC-derived NPC, generated according to the above-described spinal cord patterning protocol, were plated on a 10 cm plate at a density of 5 000 000 cells/plate and differentiated into hiPSC-MN until 60DIV. Cell culture was treated with EZ-Link Sulfo-NHS-LC-Biotin (ThermoFisher, 21335) according to manufacturer’s instructions. Briefly, cells were washed 3 times to remove amine containing media and cell debris with ice-cold PBS (pH 7.4), and then treated with biotin reagent in cold PBS buffer for a final concentration of 5 mM biotin. Plates were incubated for 30 minutes on ice to reduce active internalization of the biotin and then washed 3 times with cold PBS containing 100 mM glycine to quench and remove the excess of biotin reagent. Cells were mechanically scraped off the 10 cm plate, pelleted at 300g for 5 minutes and the pellet was resuspended in T-PER with phosphatase and protease inhibitors. The sample was then treated similar to other Western blot experiments, with sonication and ultracentrifugation to remove the insoluble lysate cell fraction. Streptavidin pulldown was performed using Dynabeads M-280 Streptavidin (Invitrogen, 11205D) on a DynaMag-2 magnet (Invitrogen, 12321D), following manufacturer’s instructions, with modification for higher elution yield (expected to be ~0.1 % of the initial cell lysates).^27^ A fraction of the initial cell lysate was used for total protein quantification. Briefly, a 20 μL volume of magnetic beads was initially washed with TPER and then mixed with 200 μL of biotinylated cell lysate containing 0.5 mg/ml proteins. The mixture was rotated overnight at 4°C. The beads were washed with TPER 4 times, and the proteins were eluted with 25 mM biotin at 95 °C for 5 minutes, followed by Laemmli 2X sample buffer (Sigma-Aldrich, S3401), at 95 °C for 10 minutes. Volumes were calculated for a final 35 µL running sample. 20 μg of the initial protein sample was used to quantify total P2X7R expression. Proteins were collected from 2 separate rounds of differentiation of the same cell line (CS8PAA) and the samples were run together.

### Vybrant CM-DiI P2X7R membrane co-localization

Vybrant CM-DiI cell labeling solution (ThermoFisher, V22888) was diluted in neuronal media and added to hiPSC-MN that had been plated onto 12 mm glass coverslips previously. Coverslips were left to incubate at 37 °C for 5 minutes followed by 3 washes in PBS. Cells were left for a post incubation period of 10 minutes (on ice) prior to PFA fixation and immunocytochemical staining using both intracellular and extracellular epitope directed P2X7R primary antibodies as previously described in this Methods section. Confocal imaging was conducted, and marker overlap was determined qualitatively using Zen 3.1 software (Zeiss, blue edition).

### Fura-2 AM calcium imaging

HiPSC-MN were passaged to glass bottom 24 well plates at a density of 50 000 cells/well and maintained in phenol red-free NDM (500µL/well). Immediately prior to calcium imaging, cells were treated with JNJ-5567 (1 mM in DMSO stock concentration) such that the final concentrations for the conditions were 3 µM or 300 nM of JNJ-5567. Controls were treated with the highest equivalent concentration of DMSO (3 µM). HiPSC-MN were bath loaded with 5 µM of Fura-2 AM (ThermoFisher, F1221) and incubated with either JNJ-5567 or DMSO, for 45 minutes at 37 °C. HiPSC-MN were washed twice in phenol red-free NDM before being left to rest in 200 µL of phenol red-free NDM for 5 minutes at 37 °C. Calcium imaging was performed on a Zeiss Axio Observer.Z1 inverted microscope equipped with a Lambda DG-4 (Sutter Instrument Company) wavelength switcher. Calcium levels were computed by determining the Fura-2 AM emission ratio with excitation at 340 nm divided by excitation at 380 nm. For all conditions, 3 coverslips of hiPSC-MN were treated with vehicle (ddH_2_O) followed by BzATP for a final concentration of 300 µM. KCl was added to all conditions following BzATP addition as a positive control for depolarization to ensure that treatment with JNJ-5567 did not prevent the normal depolarization of neurons. Traces represent average changes in Fura ratio from initial Fura ratio at t = 0 divided by initial Fura ratio (ΔF/F), corrected for background, calculated from 3 coverslips per condition, with 10-15 cells per coverslip. For the quantification of peak changes in calcium influx, Δ*F*/*F* was calculated at 30 seconds after BzATP addition in *n* = 3 technical replicates per vehicle and JNJ-treated conditions. Experiments were repeated for *n* = 3 biological replicates (cell lines CS8PAA, CS9XH7, CS0002) which were tested on independent days. All data was processed using Axiovision4 (Zeiss).

### Chronic BzATP treatment and rescue with JNJ-5567 in hiPSC-MN

#### Drug preparation

Five milligrams of BzATP (Sigma-Aldrich, B6396) was dissolved in 698.91 µL of ddH_2_O to make a 10 mM stock concentration. Ten milligrams of JNJ-5567 (Tocris Cat. 5299) was dissolved in 20.465 mL of DMSO (neat) for a stock concentration of 1 mM which was diluted in media to the working concentrations. Five mg of NAD (Roche, NAD100-RO) was dissolved in 7.5 mL of ddH_2_O to make a 1000 µM stock concentration.

#### Immunocytochemistry-based cell survival assay

HiPSC-MN were passaged to 24 well plates containing 12 mm glass coverslips as previously described in the methods section. Beginning at 60 DIV (day 0), cells were treated with either JNJ-5567 alone (1 µM), vehicle (ddH2O), BzATP (1, 10, 100 µM), or BzATP (1, 10, or 100 µM) in combination with JNJ-5567. Cells were kept in 500 µL of media which was replaced 50% each feed with drug or vehicle solutions, every third day for a total of 2 weeks. Concentrations were calculated assuming 100 µL of evaporation from each well after 3 days. For conditions without JNJ-5567, equivalent amounts of DMSO were added to each well. Similarly, volumes of dH_2_O were equalized across all conditions such that total concentrations on days 3, 6, 9, and 12 consisted of 1% v/v ddH_2_O and 0.1% v/v DMSO. On day 15, cells were fixed and stained for ChAT, Isl1, and Tuj1 following the immunocytochemistry protocol previously outlined in this Methods section. All experiments were performed with a technical replicate of *n* = 3 per condition, with *n* = 3-4 biological replicates (different control cell lines). The number of surviving ChAT^+^ cells following the 2-week treatment period were counted from images obtained from each coverslip which were analyzed using Zen 3.1 (Zeiss, blue edition) and the numbers of surviving hiPSC-MN were quantified as a percentage of surviving ChAT^+^ cells following vehicle treatment.

A similar experiment was performed to evaluate BzATP neurotoxicity in the presence of NAD^+^. The treatment conditions included: BzATP 1 or 100 µM, either with vehicle only or with 1 or 20 µM NAD^+^. The NAD^+^ dose was based on prior literature showing NAD- and dose-dependent sensitization of P2X7R in vitro astrocytes.^28^ Volumes of vehicle and drug conditions were equalized such that media contained 1% v/v ddH_2_O. This experiment was performed on a single control cell line (CS8PAA), and *n* = 3 technical replicates were considered for each treatment condition.

#### Resazurin (alamar blue)-based cell survival assay

HiPSC-MN were plated on 96-well plates (flat and treated bottom, Corning, CLS3997) at 15 000 cells/well. Beginning DIV60, cells were treated with either vehicle (DMSO and ddH2O), BzATP (1 or 100 µM) or BzATP (1, or 100 µM) in combination with JNJ-5567 (1 µM). Similar to ICC, vehicle concentrations of ddH20 and DMSO were equalized across conditions (1% v/v ddH2O and 0.1% v/v DMSO). A single control cell line (CS8PAA) was used for this experiment, and *n* = 12 wells were used for each treatment condition. Half media exchanges were performed on days 3, 6, 9 and 12, and the Resazurin assay was performed on day 15. We used AlamarBlue (AB) Cell Viability Reagent (Invitrogen, DAL1025). Briefly, based on manufacturer’s instructions,^29^ 10 µL of reagent (10% v/v) were added to each well containing 90 µL of phenol free motor neuron media, and the cells placed in the incubator, at 37 °C, for 4 hours. Additional *n* = 12 wells represented blank media with 10% resazurin or blank media without resazurin. At 4 hours, we assumed, based on the manufacturer instructions, that a plating density from 5000 to 40 000 cells/mL is expected to produce a linear reduction of resazurin. This means that even for cell survival much lower than what we saw on ICC experiments (as low as 33% of the initial density), resazurin reduction was expected to be linear with cell density. Fluorescence with 560/590 nm excitation/emission and absorbance at 570 and 600 nm were measured on a dual microplate reader. Cell viability was determined as percentage of control condition. For fluorescence-based method, after measuring FI 590 = fluorescent intensity at 590 nm emission (560 nm excitation), we calculated the % difference between treated and control cells = FI 590 of test well/FI 590 of control well × 100. For absorbance-based method, after measuring absorbance at 560 (A560) and 600 (A600) we first calculated the correction factor which accounts for blank media, R0 = (absorbance 560 in media with 10% AB − absorbance 560 of media only)/(absorbance 600 in media with 10% AB − absorbance 600 of media only), then A560 = absorbance at 560 minus the media blank at 560, and similarly A600 = absorbance at 600 minus the media blank at 600, and used this formula to calculate the % difference = A560 − (A600 × R0) for test well/A560 − (A600 × R0) for control well × 100.

## Data analyses

All statistical analyses were conducted using the GraphPad Prism 9 software. Graph bars represent mean with ±SEM and individual observations are shown as points. Statistical analyses were conducted using one-way ANOVA followed by Tukey’s test for multiple comparisons. The statistical significance was set at *P < .05, **P < .01, ***P < .001, ^****^P < 0.0001. Technical and/or biological replicates and the number of experimental conditions are indicated in the figure legends.

## Results

### P2X7R expression in human and mouse spinal cord

**Figure 1.**
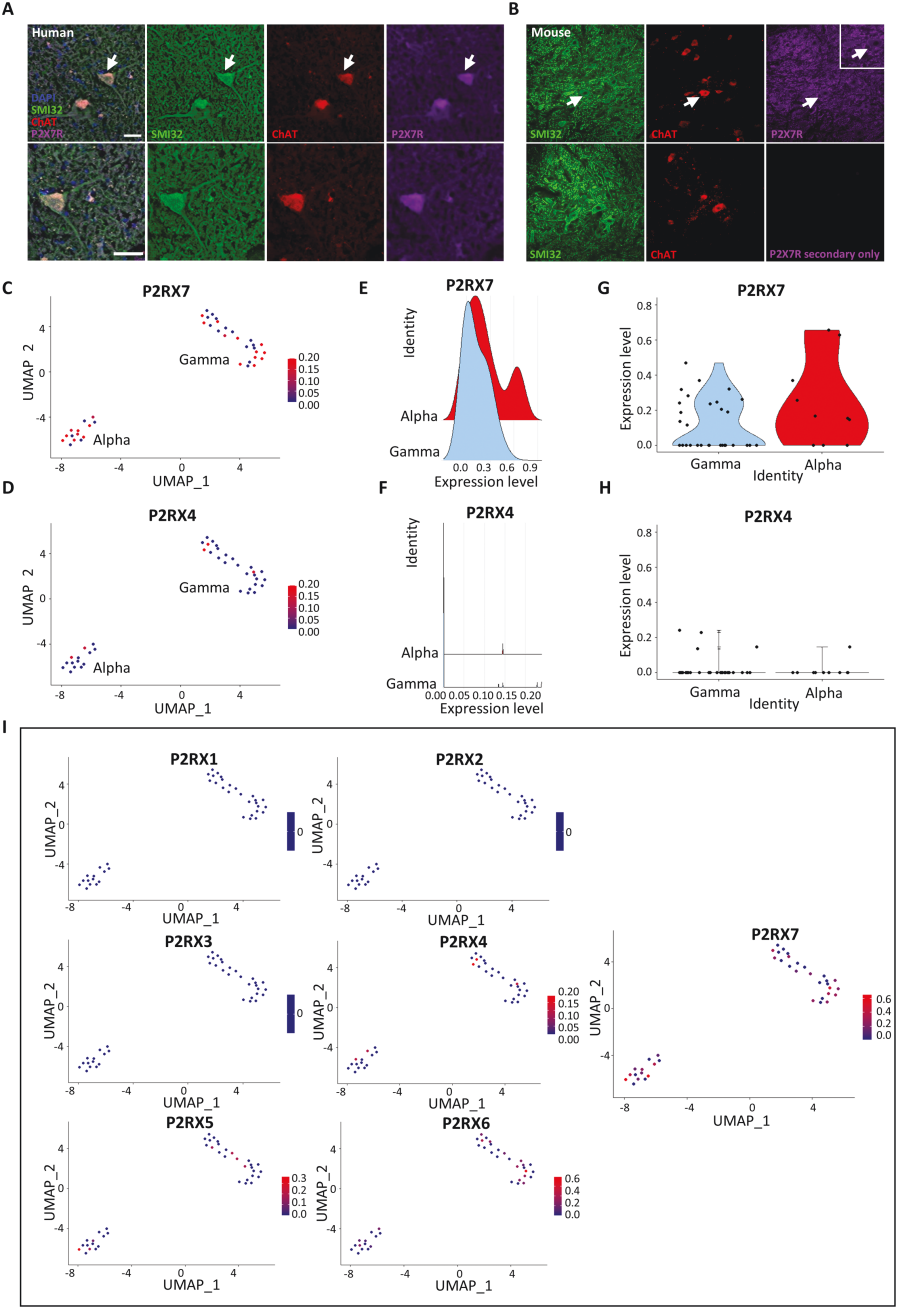
Immunohistochemical labeling and snRNA sequencing analysis showing P2X7R expression in the human cervical spinal cord. All snRNA sequencing data was collected and previously analyzed by Gautier et al., 2023. Alpha and gamma motor neuron clusters were identified by authors based on the enrichment of known marker genes from the mouse. All plots show normalized expression data. (A) Human cervical spinal cord sections showing P2X7R expression co-localized to ChAT+ MN in the ventral horn. White arrows indicate representative spinal MN that are enlarged in the lower panel. Scale bar = 50µM. (B) Mouse cervical spinal cord sections showing a similar expression pattern as observed in human with P2X7R co-localizing with ChAT+ MN in the ventral horn. (C and D) Feature plot showing expression of P2RX7 and P2RX4 in alpha and gamma motor neuron clusters from snRNA sequencing data from the human cervical spinal cord. (E and F) Ridge plots comparing P2RX7 and P2RX4 gene expression in alpha and gamma motor neurons highlighting alpha motor neurons with greater P2RX7 expression compared to gamma neurons. (G and H) Violin plots showing the distribution of motor neurons that express P2RX7 and those that express P2RX4. (I) Feature plots comparing normalized expression of all P2RX genes in human motor neurons showing that P2RX7 is expressed more strongly and in a greater proportion of motor neurons compared to all other P2RX receptors.

Our first effort to identify P2X7R on motor neurons was in human post-mortem tissue. Immunohistochemistry in human cervical spinal cord sections shows that the P2X7R strongly co-localizes with the motor neuron marker choline acetyltransferase (ChAT) in the ventral horn (**Figure 1A**). We corroborated this finding with immunohistochemistry in mouse cervical spinal cord sections which similarly showed P2X7R localization to ChAT^+^ neurons in the ventral horn (**Figure 1B**). We also noted P2X7R expression in the neuropil surrounding motor neurons which was distinct from what we observed as background staining in our secondary only control and consistent with the receptor’s known expression in glial cells (**Figure 1B**). In addition to looking at human post-mortem spinal cord tissue, we characterized P2X7R expression in human cortical tissue where P2X7R expression co-localizes with CTIP2, a marker for layer V subcerebral projection motor neurons (**Figure S1A**). We broadened our analyses to include a comparison of P2X4 receptor (P2X4R) expression in the mouse cervical spinal cord since P2X4R has been previously identified on neurons in the CNS and shares the greatest sequence homology with the P2X7R, posing the potential problem of antibody cross reactivity. We found that unlike the P2X7R, the P2X4R does not appear to be expressed in motor neurons but is diffusely expressed throughout the cervical spinal cord (**Figure S2A**). To examine whether the pattern of expression we observed for P2X4R was the result of the receptor’s localization to another cell type, we compared P2X4R expression in mouse cervical spinal cord sections from a disease model whose phenotype at endstage is characterized by microglial and astrocytic infiltration into the ventral horn. While the P2X4R does not strongly localize to ChAT^+^ motor neurons, it is distinctly expressed in astrocytes and microglia, co-localizing with GFAP^+^ and CD68^+^ cells respectively (**Figure S2B**). The expression of P2X7R in motor neurons in both cervical spinal cord tissue and cortical tissue, as well as its distinct expression pattern compared to the P2X4R suggests that the P2X7R is expressed in human neurons in vivo and that the P2X7R may contribute to the susceptibility of motor neurons to insult from increased extracellular ATP.

### P2X7R transcripts in human spinal MN using snRNA sequencing datasets

To complement the qualitative analyses, we sought to quantify the expression of the P2X7R in human motor neurons in vivo by analyzing a single nucleus RNA sequencing dataset (snRNA) that was generated from control donor tissues from the cervical spinal cord by Gautier et al.^18,19^ Motor neuron populations were identified by based on the enrichment of motor neuron-specific markers identified in humans and mice. When P2X7R expression is compared to P2X4R expression, we found that P2X7R is more strongly expressed in a greater proportion of motor neurons compared to P2X4R (Figure 1C-H). We validated this result in a separate snRNA sequencing dataset generated from the human lumbar spinal cord by Yadav et al.,^18^ and similarly found that the P2X7R is more strongly expressed in motor neurons of the human lumbar spinal cord compared to P2X4R (**Figure S3A**) and that the P2X4R is most strongly expressed in microglia relative to its expression in other cell clusters (**Figure S3B**). This reflects the immunohistochemical observations in the mouse and human cervical spinal cord which show that while the P2X7R is expressed in ChAT^+^ motor neurons, the P2X4R appears to be exclusively localized to non-neuronal GFAP^+^ astrocytes and CD68^+^ microglia cells. Using ridge plots and violin plots of normalized count data from the cervical spinal cord, when motor neurons are further subdivided into alpha and gamma subtypes, the P2X7R appears to be expressed in both alpha and gamma subtypes while the P2X4R is expressed at very low levels in both (Figure 1E-H). Furthermore, a greater proportion of alpha motor neurons express higher levels of P2X7R compared to gamma motor neurons potentially suggesting a selective susceptibility of alpha motor neurons to increased extracellular ATP compared to gamma motor neurons (**Figure 1E and F**). When examining the expression of all other P2X receptors in cervical spinal cord motor neurons, not all P2X receptors are present, confirming what has been previously determined about the cellular localization of these receptors to cell types that are not present in the CNS.^30^ Additionally, the P2X7R is expressed in the greatest proportion of motor neurons compared to all other P2X subtypes suggesting that P2X7R is the most relevant purinergic receptor to target as part of our proposed mechanism of ATP-mediated motor neuron toxicity (**Figure 1I**). We observed this same pattern of expression in motor neurons from the human lumbar spinal cord (**Figure S3C**). Motor neuron subtype vulnerability to cell death has been noted in neurodegenerative disorders like ALS, where oculomotor and Onuf’s nuclei are relatively spared when compared to spinal motor neurons. To assess whether the P2X7R is expressed in greater proportions among these populations, we examined an RNA sequencing dataset generated from laser captured spinal cord, oculomotor, and Onuf’s nucleus motor neurons from human post-mortem tissue (GEO accession: GSE93939)and show that RNA transcripts of the P2X7R and, to a lesser degree the P2X4R, are present at similar levels amongst these neuron subtypes suggesting that the presence of the P2X7R alone is likely not deterministic of selective vulnerability (**Figure S4A**). For context, the expression of known calcium binding proteins which are believed to contribute to a relative resistance to death are also detailed (**Figure S4B**). These sequencing analyses demonstrate that the presence of the P2X7R in human motor neurons is consistent between datasets, across 2 different regions of the human spinal cord, and suggest that the differential expression of P2X7R compared to P2X4R is consistent with the immunohistochemical staining in human and mouse cervical spinal cord.

### P2X7R are expressed in hiPSC-derived spinal motor neurons

**Figure 2.**
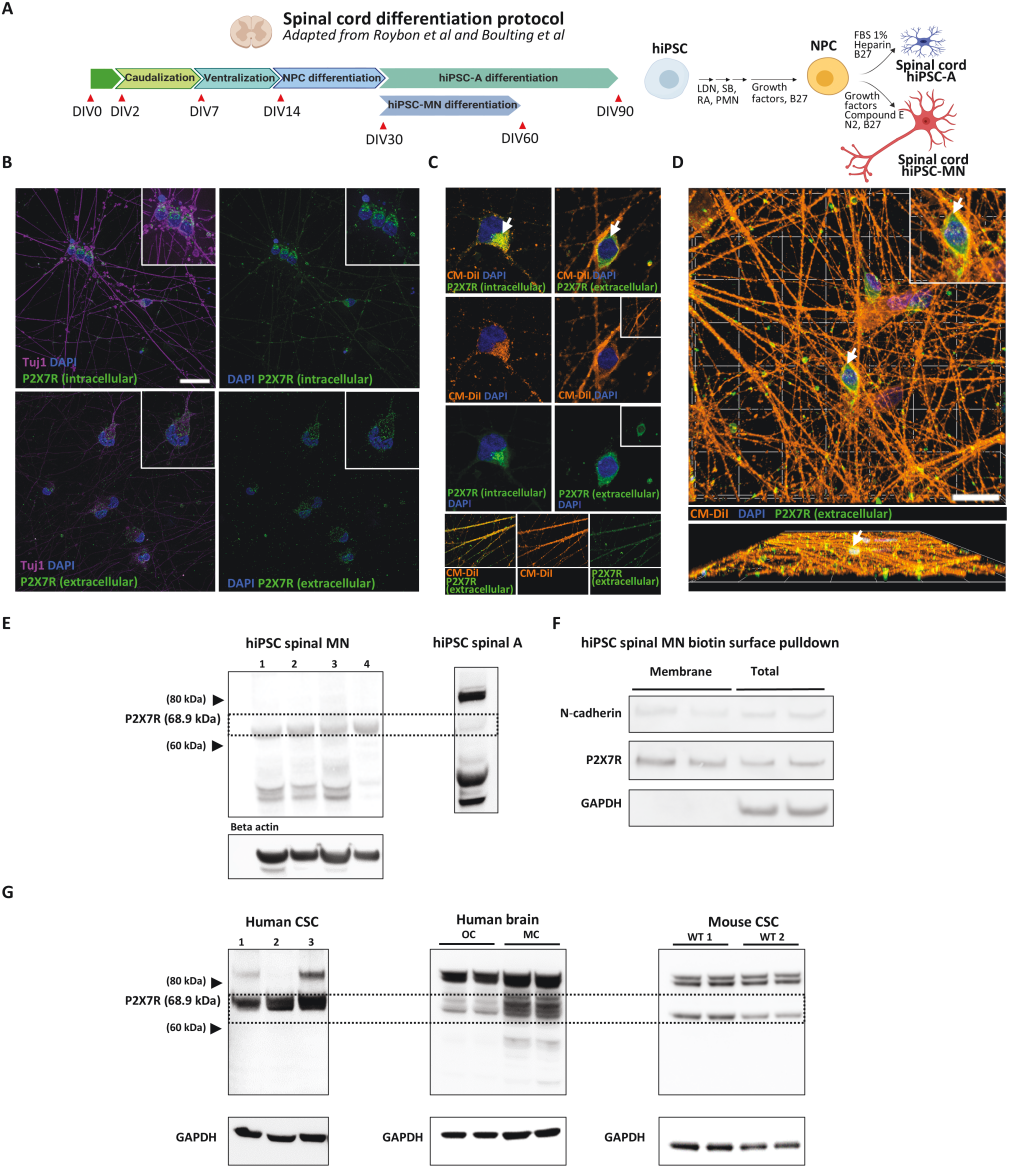
P2X7R expression on spinal hiPSC-MN with intracellular compared to extracellular epitope-directed antibodies. (A) Graphical depiction of the differentiation protocol used to generate spinal motor neurons from hiPSC. (B) Immunohistochemical labeling of spinal hiPSC-MN with P2X7R antibodies directed to either an intracellular or extracellular portion of P2X7R showing differential staining patterns. Scale bar = 20 µm. (C) Confocal imaging comparing extracellular and intracellular epitope-directed P2X7R antibodies relative to DiI-labeled membranes on hiPSC-MN. (D) Confocal 3D rendering of CM-DiI labeled hiPSC-MN showing colocalization of P2X7R (extracellular epitope) with DiI-positive areas on the cell body (white arrow) and the neurites. Scale bar = 50 µm. Detail of confocal imaging showing co-localization of punctate P2X7R (extracellular epitope) expression along hiPSC-MN neurites. (E) Western blots showing P2X7R expression in hiPSC-MN, hiPSC-spinal astrocytes. Samples 1-4 represent distinct hiPSC lines from control subjects. (F) The membrane protein fraction of hiPSC-MN after streptavidin-biotin pulldown contains P2X7R (marked by extracellular P2X7R antibody) and membrane N-Cadherin but not cytoplasmic GAPDH. Each band represents a separate protein sample from the CS8PAA hiPSC line. (G) P2X7R expression in human cervical spinal cord (CSC), human brain and mouse cervical spinal cord. All western blots were conducted using the intracellular epitope-directed P2X7R antibody.

Mature hiPSC-MN (**Figure 2A**) were differentiated using a protocol that generates relatively pure ChAT^+^ alpha motor neurons without astrocyte contamination (**Figure S5A****).** HiPSC-MN were stained for P2X7R using antibodies directed to an intracellular or extracellular epitope of the receptor to differentiate between intracellular and surface expression. As expected, hiPSC-MN stained with the intracellular epitope-directed P2X7R antibody showed P2X7R expression that was localized within the cytoplasm but not the nuclei of hiPSC-MN (**Figure 2B**). Importantly, to demonstrate that the extracellular epitope of P2X7R is present on the surface of hiPSC-MN, cells were not permeabilized prior to staining for the extracellular epitope. As expected, the extracellular epitope-directed P2X7R antibody showed a differential staining pattern compared to that of the intracellular epitope-directed antibody, having more punctate expression and bright foci on the cell body and neurites of neurons (**Figure 2B**). The localization of P2X7R that was seen in spinal hiPSC-MN was also demonstrated in hiPSC-derived CTIP2^+^ cortical neurons (**Figure S1B**) which were differentiated following a protocol outlined in **Figure S5B**. To further confirm that the P2X7R is trafficked to the membrane of hiPSC-MN, cell membranes were labeled with Vybrant CM-DiI prior to the detection of extracellular or intracellular portions of the receptor. Localization of P2X7R relative to the cell membrane boundaries was as expected, with the extracellular portion of P2X7R overlapping with CM-DiI positive areas of the cell while the intracellular portion of P2X7R was contained within the boundary of CM-DiI (**Figure 2C**). While the extracellular epitope of P2X7R shows some overlap on the cell body of neurons (**Figure 2C**), greater overlap was observed on neurites (**Figure 2C**). This is particularly apparent in the 3D visualization of hiPSC-MN with CM-DiI which shows punctate P2X7R expression that co-localizes with CM-DiI (**Figure 2D**).

### hiPSC-MN P2X7R expression mirrors that from mouse and human spinal cord

To validate the immunocytochemical findings as well as identify differential expression patterns between human and mouse, P2X7R protein expression was examined in 4 hiPSC-MN lines and compared to human iPSC-derived astrocytes (hiPSC-A), human occipital cortex, human motor cortex, and human cervical spinal cord sections (CSC), as well as mouse spinal cord sections by Western blot (**Figure 2E and G**). Localization of the P2X7R in the membrane fraction of hiPSC-MN was demonstrated using streptavidin-biotin pulldown (**Figure 2F**). The full-length 68.9 kDa P2X7R band is observed in all hiPSC-MN lines and to a lesser degree, hiPSC-A, and reflects a similar pattern of expression to human occipital and motor cortex and CSC samples suggesting that P2X7R protein expression in vitro reflects protein expression in vivo. In addition, multiple smaller bands in the hiPSC-MN samples were attributed to alternative splice variants or subunit fractionation. A consistently heavier band than the full-length receptor was noted and may correspond to a modified form of the receptor present in astroglia since the band is present in hiPSC-A, human cervical spinal cord, human brain, and mouse spinal cord but not in hiPSC-MN alone.

### BzATP induces calcium influx into hiPSC-MN via P2X7R activation

**Figure 3.**
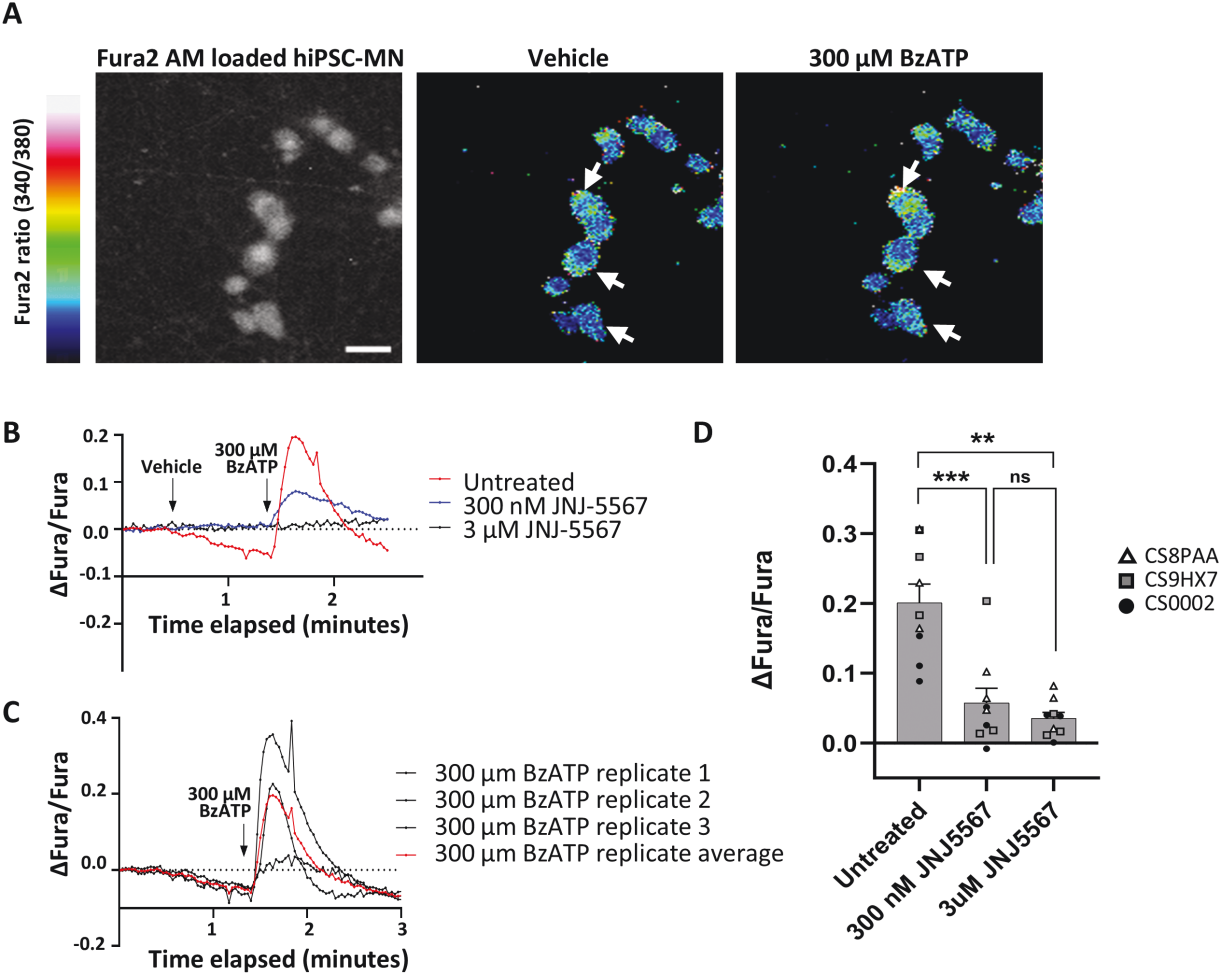
Treatment of control hiPSC-MN with BzATP leads to P2X7R-mediated calcium influx blocked by JNJ-5567. (A) Colorimetric image showing the change in Fura-2 AM ratio following the application of 300 µM of BzATP compared to vehicle. Scale bar = 20 µm. (B) Fura-2 AM signal in response to 300 µM of BzATP following prior incubation in either 3 µM or 300 nM of JNJ-5567 showing a reduction in calcium influx that is dependent on the concentration of JNJ-5567. (C) Representative Fura-2 AM signals from 3 technical replicates from a single hiPSC-MN cell line treated with 300 µM of BzATP showing a stereotypic response. (D) Quantification of the maximum change in intracellular calcium following BzATP application for the experiments shown in (B). Significance values indicate ^**^P < 0.01, ^*^^**^P < 0.001, *n* = 3 biological replicates (cell lines CS8PAA, CS9XH7, CS0002) and *n* = 3 technical replicates per condition.

P2X7R is an ionotropic, calcium permeable, cation channel that allows calcium influx to be used as a functional correlate of the receptor’s activation on the surface of hiPSC-MN. To investigate the function of P2X7R in hiPSC-MN, we opted for a pharmacological approach that relies on a potent and selective P2X7R antagonist developed by Janssen Pharmaceuticals, JNJ-47965567 (JNJ-5567) to selectively block the P2X7R prior to treatment with an ATP agonist, BzATP. BzATP is the most potent ATP agonist that selectively activates P2X7R and as such, is most widely used in functional studies. For calcium imaging, we chose a concentration of BzATP that was high enough to have an acute effect given its diffusion throughout the media in the well. Using Fura-2-AM ratiometric calcium imaging, we demonstrate that 300 µM of BzATP applied acutely to hiPSC-MN triggers the influx of calcium into cells (Figure 3A-C).This effect can be blocked if cells are incubated for 45 minutes in 3 µM JNJ-5567 (**Figure 3B and D**). Prior incubation of hiPSC-MN with 300 nM JNJ-5567 attenuates calcium influx but does not fully eliminate it, suggesting a dose-dependent effect of the drug antagonist on calcium influx and specificity of the antagonist in blocking calcium influx through P2X7R.

### P2X7R activation induces motor neuron cell death and is rescued by P2X7R antagonist

**Figure 4.**
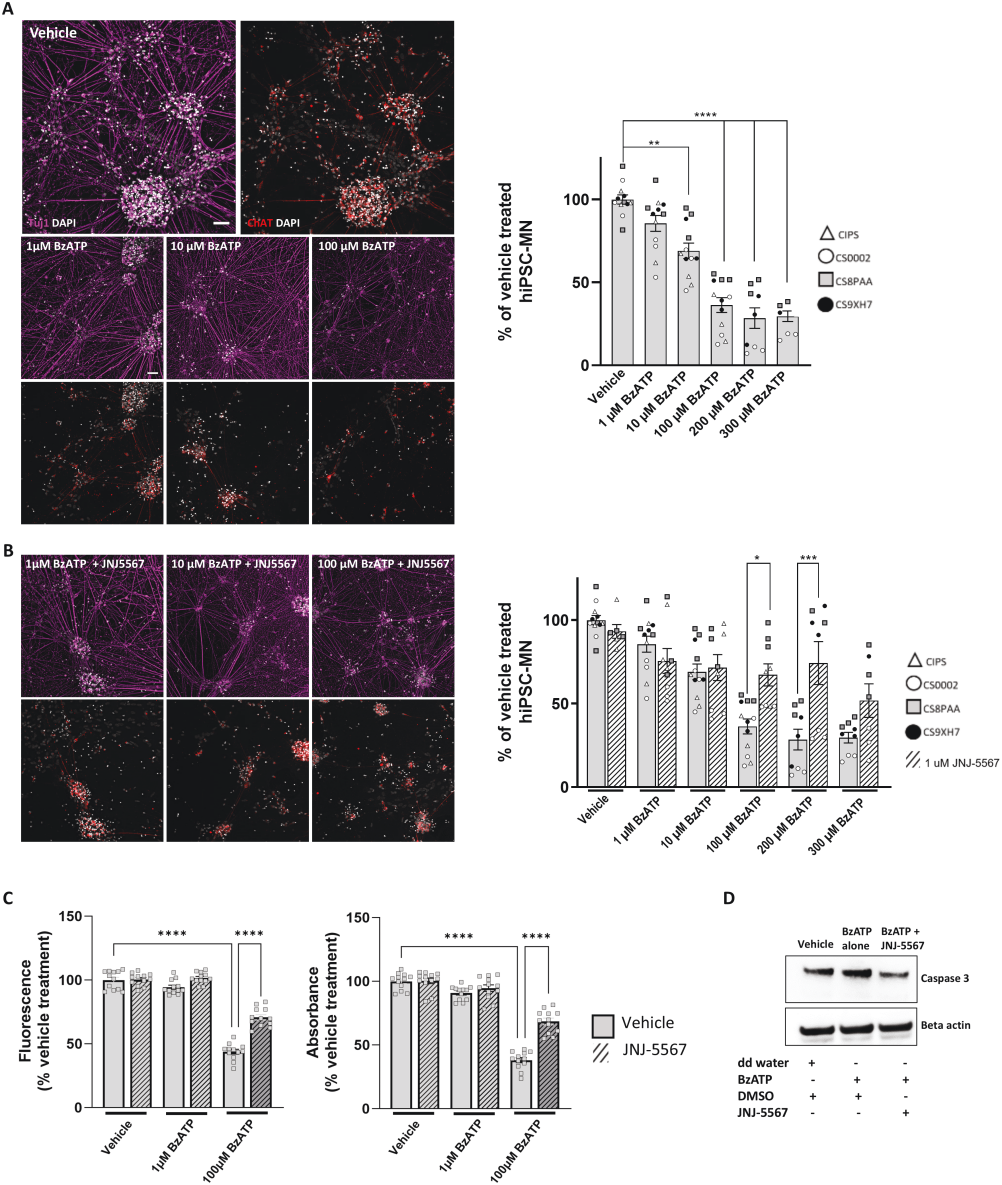
Treatment of control hiPSC-MN with BzATP leads to P2X7R-mediated, dose dependent, caspase-mediated, motor neuron loss rescued by by JNJ-5567. (A) Immunocytochemistry and motor motor neuron quantification showing survival of hiPSC-MN treated with BzATP for 2 weeks. (B) The P2X7R antagonist JNJ-5567 provides neuroprotection of hiPSC-MN following BzATP incubation. Scale bar = 50 µm. (C) Neurotoxicity by BzATP and neuroprotection by JNJ-5567 were confirmed using alamarBlue (resazurin) assay, where fluorescence and absorbance of treated and untreated conditions where compared to vehicle condition. (D) Western blot results showing increased caspase 3 expression in hiPSC-MN treated for 2 hours in 1 mM of BzATP which could be reduced with prior incubation in 3 µM of JNJ-5567. Significance values indicate *P < .05, **P < .01, ***P < .001, ^****^P < .0001. For (A) and (B) *n* = 4 biological replicates represented as different shaped data points, and *n* = 3 technical replicates per condition. For (C), a single cell line (CS8PAA) and *n* = 12 technical replicates were used.

To test whether increased extracellular ATP directly leads to hiPSC-MN toxicity, mature hiPSC-MN (DIV60) were treated with increasing concentrations of BzATP or vehicle (ddH_2_O), for 2 weeks. BzATP or vehicle was added in concentrations of 1, 10, 100, 200, and 300 µM every third day via half media exchanges, after which cells were fixed and stained for immunohistochemistry in order to calculate the percentage of surviving motor neurons following treatment. BzATP is 10 times more potent than ATP so the concentrations of BzATP used represent in this experiment represent a titration to allow for the observation of the full spectrum of response of P2X7R activation, rather than just the maximal response. After 2 weeks, hiPSC-MN that were treated with 100, 200, and 300 µM BzATP showed significant, dose-dependent ChAT^+^ motor neuron loss, suggesting that MN loss is a function of increasing ATP concentration (**Figure 4A**). In a separate experiment, hiPSC-MN were treated with increasing concentrations of BzATP or vehicle, in combination with either 1 µM of the P2X7R-specific antagonist JNJ-5567 or DMSO, to determine whether blocking activation of P2X7R on motor neurons is neuroprotective in the context of chronically elevated extracellular ATP. We found that 1 µM JNJ-5567 administered in conjunction with BzATP, significantly rescued the percentage of ChAT^+^ hiPSC-MN following the two-week treatment period in the conditions of 100 µM and 200 µM BzATP, compared to hiPSC-MN that were treated with an equivalent concentration of DMSO, indicating that blocking P2X7R activation confers neuroprotection in conditions of elevated extracellular ATP (**Figure 4B**). We additionally preformed a resazurin live cell assay to quantify BzATP induced neurotoxicity as a function of change in absorbance and fluorescence by resorufin, a pink fluorescent protein that is converted from resazurin by redox reactions of metabolically active cells. In agreement with our immunocytochemical results, we found that hiPSC-MN treated with 100 µM of BzATP for 2 weeks resulted in significantly reduced cell viability compared to hiPSC-MN treated with vehicle or 1µM BzATP, and that this neurotoxicity can be significantly rescued through the application of JNJ-5567 (**Figure 4C**).

### P2X7R activation leads to changes in pro-apoptotic markers

While having observed cell death as a result of increasing BzATP concentrations by immunocytochemical and biochemical analyses, we sought to identify changes in intracellular signalling cascades that contribute to BzATP- induced death of hiPSC-MN using western blot (**Figure 4D**). HiPSC-MN treated with 1 mM of BzATP for 2 hours followed by a 3-hour post incubation period resulted in increased caspase 3 expression compared to hiPSC-MN treated with vehicle, which could be reduced by prior incubation in JNJ-5567 (3 µM). This suggests that acute treatment with BzATP is enough to directly cause hiPSC-MN to undergo cell death via a caspase 3-mediated process that can be blocked with JNJ-5567.

### P2X7R is not sensitized by NAD-dependent ADP ribosylation in hiPSC-MN cultures

Changes in P2X7R gating that lower its activation threshold, including NAD-dependent ADP ribosylation by ecto-ADP-ribosyltransferase 2 (ARTC2), have been proposed.^28,31–33^ To investigate whether this mechanism could be sensitize P2X7R activation in this hiPSC-MN model, we first assessed ARTC2 protein expression in hiPSC-MN cultures. We found that ARTC2 was absent in hiPSC-MN and only minimally expressed in hiPSC-A cultures (**Figure 6A**). Subsequently, we conducted an immunocytochemistry-based cell survival assay on hiPSC-MN cultures and found no neurotoxic effects from low-dose BzATP (1 µM) in the presence of NAD^+^ (**Figure S6B**). Together these results suggest that in this hiPSC-MN culture system, ARTC2 does not significantly sensitize MN to ATP-induced cell death.

## Discussion

This work demonstrates a mechanism by which motor neurons undergo cell death as a result of extracellular ATP binding directly to P2X7R on their surface. The P2X7R represents a unique pharmacological target because its activation threshold is significantly higher than all other P2X family members, having an EC_50_ value of 100 µM.^6^ This suggests that the P2X7R functions as a silent receptor that is only active in pathological states marked by increased extracellular ATP. This is in contrast to other members of the P2X receptor family, like P2X4R, which despite sharing the greatest sequence homology with P2X7R, has an activation threshold ten times lower, with an EC_50_ value of only 10 µM.^6^ We investigated the distribution of P2X7R relative to P2X4R on motor neurons because they both function as non-specific cation channels and have both been reportedly expressed by neurons in the CNS.^34,35^ We found that P2X4R is expressed in ChAT^+^ motor neurons and the surrounding neuropil but is enriched in non-neuronal cell types including CD68^+^ microglia and GFAP^+^ astrocytes. In comparison, P2X7R is expressed in ChAT^+^ motor neurons in both human and mouse cervical spinal cord tissue. We corroborated these observations with analyses of 2 separate single nucleus RNA sequencing datasets from the cervical and lumbar regions of the human spinal cord and show that P2X7R has much higher expression in a larger proportion of motor neurons compared to all other P2X receptors in both anatomical regions, while P2X4R is more strongly expressed in non-neuronal cells like microglia.

The idea that non-neuronal cells contribute to changes in extracellular ATP has been previously explored, particularly as it relates to the neuroinflammatory cascade. As a pro-inflammatory molecule, ATP is well known to be released from damaged or dying cells, subsequently binding to pattern recognition receptors on microglia and leading to the induction of the NLRP3 inflammasome.^14^ The cytosolic concentration of ATP has been estimated to be on the order of 3-10 mM at steady state suggesting that if cell membrane integrity is compromised due to a variety of possible insults, sufficient concentrations of ATP would be released to activate high threshold purine receptors as a damage associated molecular pattern (DAMP).^36^ Astrocytes participate in reciprocal interactions with microglia, making them important players in the neuroinflammatory cascade. ATP is known to potentiate pro-inflammatory signaling through autocrine feedback on astrocytes whereby ATP mediates further ATP release.^37^ This, in turn, enhances IL-1β-induced NFkB and AP-1 activation and leads to increased chemokine production including IL-8 and monocyte chemoattractant protein 1 (MCP1).^2,37,38^ Astrocytes are also positioned to act as significant modulators of synaptic function and transmission as well as regulators of neuronal health given that they comprise a “tripartite” synapse with the presynaptic and postsynaptic membranes of neurons which facilitates paracrine signaling.^39^ Our group has previously reported that ATP is one of the molecules that is released through connexin 43 hemichannels of ALS hiPSC-derived astrocytes independent of inflammatory stimulation, suggesting that there are multiple pathways that can contribute to increased ATP in the extracellular milieu and directly impact motor neuron survival.^40^ Using an hiPSC-MN platform, we show that increasing concentrations of BzATP lead to a dose dependent loss of ChAT^+^ MN following a chronic treatment period of 2 weeks, the same time point used to observe significant astrocyte mediated neurotoxicity described in previous work.^40^ hiPSC-MN survival is significantly rescued by JNJ-5567 when administered concurrently with BzATP, suggesting that the rescue effect was specific to the blockade of P2X7R and that chronic activation of P2X7R in motor neurons otherwise leads to cell death.

Following acute BzATP treatment, caspase 3 is increased in hiPSC-MN and corresponds to a transient increase in intracellular calcium, suggesting that MN death is in part mediated by the influx of calcium. These findings agree with what has been found using murine models which demonstrate that BzATP can induce caspase-3 mediated cell death in embryonically derived rat spinal cord motor neurons and that calcium chelation effectively rescues survival following BzATP treatment.^10^ Calcium is a potent secondary messenger and is kept within a strict concentration range in the cell due to its potential to disrupt a variety of structural and downstream signaling processes. Motor neurons are particularly susceptible to calcium overload due to their inherent low levels of calcium buffering proteins like parvalbumin and calbindin compared to other neuron types^41^ We extended our analysis on the expression of P2X7R to include other motor neuron populations, that have been shown to be selectively resistant to cell death both pathologically and clinically in ALS, specifically: the oculomotor and Onuf’s nucleus motor neurons. We appreciated RNA transcripts of P2X7R and, to a lesser degree P2X4R, in both neuron subtypes but did not see a significant difference to suggest that the absence of P2X7R expression in these motor neuron subtypes alone would account for their relative resistance to death. We postulate that the presence or absence of P2X7R is likely not the primary determinant of selective susceptibility to degeneration, but rather a strong contributing factor due to the increased likelihood of calcium overload in response to persistent activation. This is a particularly relevant mechanism of cell death since it has been previously shown that the P2X7R does not inactivate following prolonged stimulation with ATP but rather leads to the formation of a macropore that not only destabilizes the cell membrane but indiscriminately allows for the continued passage of ions like calcium as well as other potentially toxic factors.^7,42^

Changes in P2X7R gating that decrease its activation threshold, have potential implications in pathology given our finding that chronic P2X7R activation leads to cell death and in addition, may allow for ATP-induced neurodegeneration to occur at extracellular ATP levels similar to those observed under physiological conditions in vivo. Studies by Hubert et al^43^ and Hirayama et al^28^ identified ADP-ribosylation mediated by ecto-ADP-ribosyltransferase 2 (ARTC2) as a mechanism of post-translational modification of P2X7R which may sensitize it to low extracellular ATP concentrations. In a cerebral ischemia study, NAD^+^, a substrate for ARTC2, lowered the activation threshold of P2X7R in dose-dependent manner in astrocytes.^28^ We tested whether this mechanism could be translated to our platform but did not find any effects of NAD^+^ on ATP-induced neurotoxicity. This likely reflects the lack of expression of ARTC2 that we observed in our hiPSC-MN, supporting limited prior literature showing that this enzyme is mainly expressed in in astrocytes and microglia.^28,44^ However, this mechanism could be relevant for in vitro systems where hiPSC-A, which we found to express ARTC2, are co-cultured with neurons as well as in vivo in ALS, where it may be involved in non-cell autonomous neurotoxicity. Similar research lead Robinson et al to demonstrate that P2X7R activation is modulated by cholesterol binding in the proximal C-terminal, which is uniquely long compared to other P2X subtypes and is thought to coordinate many other intracellular signaling cascades.^45^ This could be particularly relevant in human motor neuron pathology, and specifically ALS, where elevated serum cholesterol levels have been identified as a risk factor in large epidemiological and genetic studies.^46^

Having validated P2X7R expression in hiPSC-MN, we are able to explore P2X7R antagonism as a possible therapeutic strategy using a human platform of relatively pure motor neuron populations. Given the previous failures in translation of compounds that have demonstrated efficacy in transgenic mouse models, especially in models of MN diseases like ALS where most cases lack a genetic basis,^47,48^ it is crucial to acknowledge the limitations of solely relying on murine models to evaluate novel therapeutic targets. Previous studies that have investigated P2X7R antagonism with JNJ-5567 and other P2X7R antagonists in the SOD1 mouse model of ALS show conflicting results in part due to differences in P2X7R antagonist dose regimens, treatment start time relative to disease progression and poor central nervous system bioavailability. Furthermore, it has been established that there are functional differences between mouse and human P2X7R that lead to differential antagonist specificity, receptor inactivation speeds and agonist potency^5-7^ which become particularly relevant considerations in the evaluation of a novel drug. In addition to these functional differences, there are also structural differences between mouse and human P2X7 receptors owing to the significant alternative splicing that generate species-specific P2X7R isoforms that have been shown to have distinct roles in processes including cell growth, cell death and inflammation.^11^ JNJ-5567 is part of a series of compounds that includes JNJ-54175446 (JNJ-5446) which, like JNJ-5567, has high specificity for P2X7R and high^49^ blood brain barrier permeability, but has additionally been proven safe and tolerable suggesting that prolonged P2X7R antagonism does not interfere with physiological functions in a way that causes significant adverse effects. JNJ-5446 is currently under investigation in a phase 2 clinical trial for major depressive disorder (MDD) (clinicaltrials.gov: NCT04116606) however, while the translational potential of P2X7R antagonism has begun to be evaluated for neuropsychiatric indications, it has not been evaluated in motor neuron disease. By using JNJ-5567, we are able to evaluate the specific contribution of P2X7R blockade on functional correlates of the receptor’s expression in human motor neurons and provide insight into the translational potential of JNJ-5446 for indications beyond MDD, including motor neuron diseases. This work broadens our understanding of purinergic signaling to one that includes extracellular ATP as a direct precipitator of motor neuron death at elevated concentrations, like those observed in pathological conditions marked by increased inflammation. This suggests that the activation of P2X7R by elevated levels of extracellular ATP represents a point of convergence between neuroinflammatory cascades and motor neuron death, making the receptor a unique pharmacological target that is translationally relevant and that could potentially allow for a combinatorial approach which would serve to both dampen inflammation as well as provide a direct neuroprotective benefit.

## Supplementary material

Supplementary material is available at *Stem Cells Translational Medicine* online.

szae074_suppl_Supplementary_Figures_S1-S6

## Data Availability

All study data are included in the main text and SI Appendix.
