# Supplementary material for: Exploring P2X7 receptor antagonism as a therapeutic target for neuroprotection in an hiPSC motor neuron model
Source: Stem Cells Transl Med. 2024 Oct 17;13(12):1198–212. doi: 10.1093/stcltm/szae074 (PMC11631223; doi:10.1093/stcltm/szae074)

Supplementary Figure S1. Immunohistochemical labeling of human motor cortex and hiPSC-derived human cortical neurons. A) Human motor cortex sections showing P2X7R colocalization with CTIP2, a marker for subcerebral projection motor neurons found in layer V of the cortex. Scale bar=10µm. **B)** Human iPSC-derived corticospinal motor neurons showing a similar pattern of P2X7R expression co-localized with CTIP2 positive neurons, *in vitro.* P2X7R colocalizes with CTIP2 but is not exclusive to CTIP2+ neurons. White arrows indicate representative corticospinal MN. Scale bar=50µm.

Supplementary Figure S2. P2X4R expression in the mouse cervical spinal cord. A) Immunohistochemical labeling of cervical spinal cord sections from wildtype (B6SJL) compared to SOD1G93A mice showing P2X4R upregulation at endstage. **B)** Immunohistochemical labeling of mouse cervical spinal cord showing P2X4R upregulation occurs in $GFAP^{+}$ astrocytes and $CD68^{+}$ microglia at disease endstage. Scale bar=50µm. White arrows indicate representative motor neurons, microglia, and astrocytes.

Supplementary Figure S3. **Analysis of a single nucleus RNA sequencing dataset from the human lumbar spinal cord showing differential P2X7 and P2X4 expression in spinal motor neurons**. All plots are generated from single nucleus RNA sequencing data that was originally collected by Yadav et al., 2023. Clusters were manually annotated by authors based on the enrichment of known markers for each identified cell type. **A&B)** Feature plots showing the distribution of P2X7 and P2X4 expression across all cell types in the human lumbar spinal cord. **C)** Feature plots comparing the expression of all P2X receptors in human lumbar motor neurons. P2X7 appears to be expressed at a higher level in a greater proportion of motor neurons in the human lumbar spinal cord compared to all other P2X receptors

Supplementary Figure S4. **RNA sequencing analysis of laser captured nuclei from human lumbar motor neurons, Onuf's nucleus and oculomotor neurons. A)** RNA sequencing analysis of P2RX7 and P2RX4 genes. P2RX7 is enriched in lumbar spinal cord (SC) motor neurons, but is also expressed in Onuf's nucleus and oculomotor neurons. The expression of P2X4R is minimal across the different neuronal populations. **B)** Comparison of expression of calcium binding genes Parvalbumin (PVALB), calbindin (CALB1) and calretinin (CALB2). Oculomotor neurons (OMN), which are resistant to degeneration in ALS, express high levels of calcium binding genes. Values represent LogNormalized raw counts stored found under the GSE accession number: GSE93939.

Supplementary Figure S5. Regionally-specific differentiation protocols to generate human-induced pluripotent stem cell-derived neurons (hiPSC-MN) and astrocytes (hiPSC-A). A) The spinal cord patterning protocol generates $ChAT^{+}$ motor neurons (MN) in 60 days and GFAP astrocytes in 90 days, as shown in the representative immunofluorescence images. Given hiPSC-MN cultures are treated with ARA-C prior to plating, they show minimal contamination from GFAP astrocytes, whose proportion is not influenced by BzATP treatment. **B)** The cortical patterning protocol generates a population of CTIP2 corticospinal MN. Abbreviations: LDN, LDN193189; FBS, fetal bovine serum; KSR, knockout serum replacement RA, retinoic acid; SB, SB431542; PMN, purmorphamine.

Supplementary Figure S6. **P2X7R is not sensitized by NAD-dependent ADP ribosylation in hiPSC-MN cultures.** **A)** Western blot analysis of ecto-ADP-ribosyltransferase 2 (ARTC2) in hiPSC-MN and hiPSC-A shows significant expression in astrocytes only. Each band represents a distict cell line (CS8PAA and C9XH7). **B)** The effects of different doses of bzATP (0, 1 and 100µM) on the survival of $CHAT^+$ MN were evaluated in the presence of increasing doses of NAD (1µM and 20µM) and compared to vehicle treatment. NAD+ does not affect the neruotoxicity of BzATP, which occurred at 100µM but not at 1µM. Significance values indicate *$p < 0.05$, **$p < 0.01$, ***$p < 0.001$, ****$p < 0.0001$. A single cell line (CS8PAA) was used in this experiment, and n=3 cover slips per condition. Scale bar =50µm

Supplementary 1

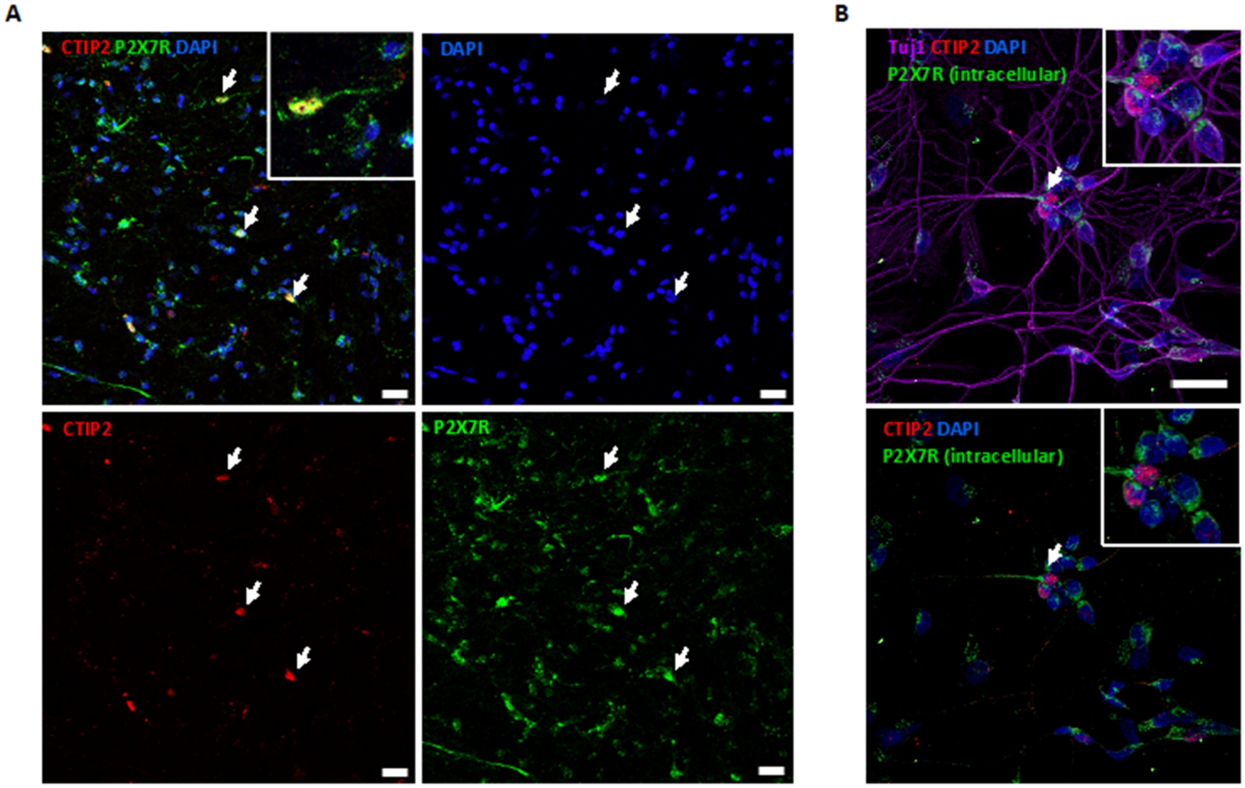

Supplementary 2

A

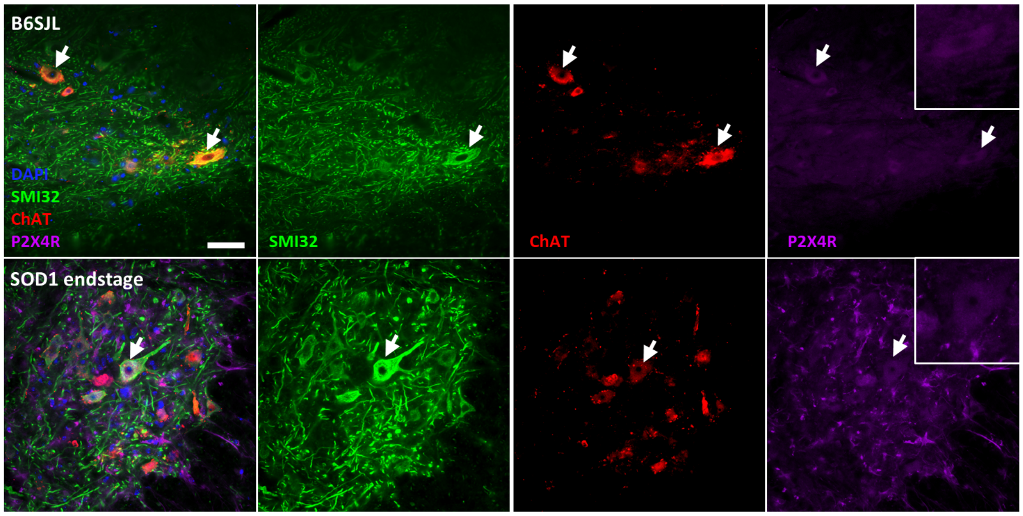

B

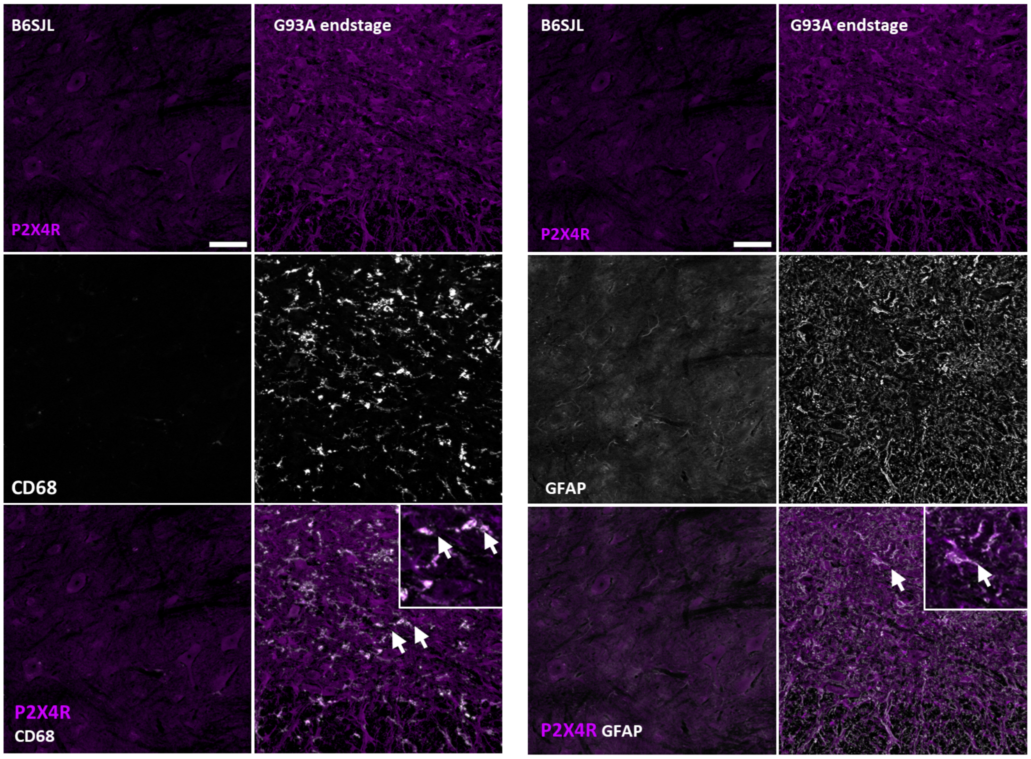

Supplementary 3

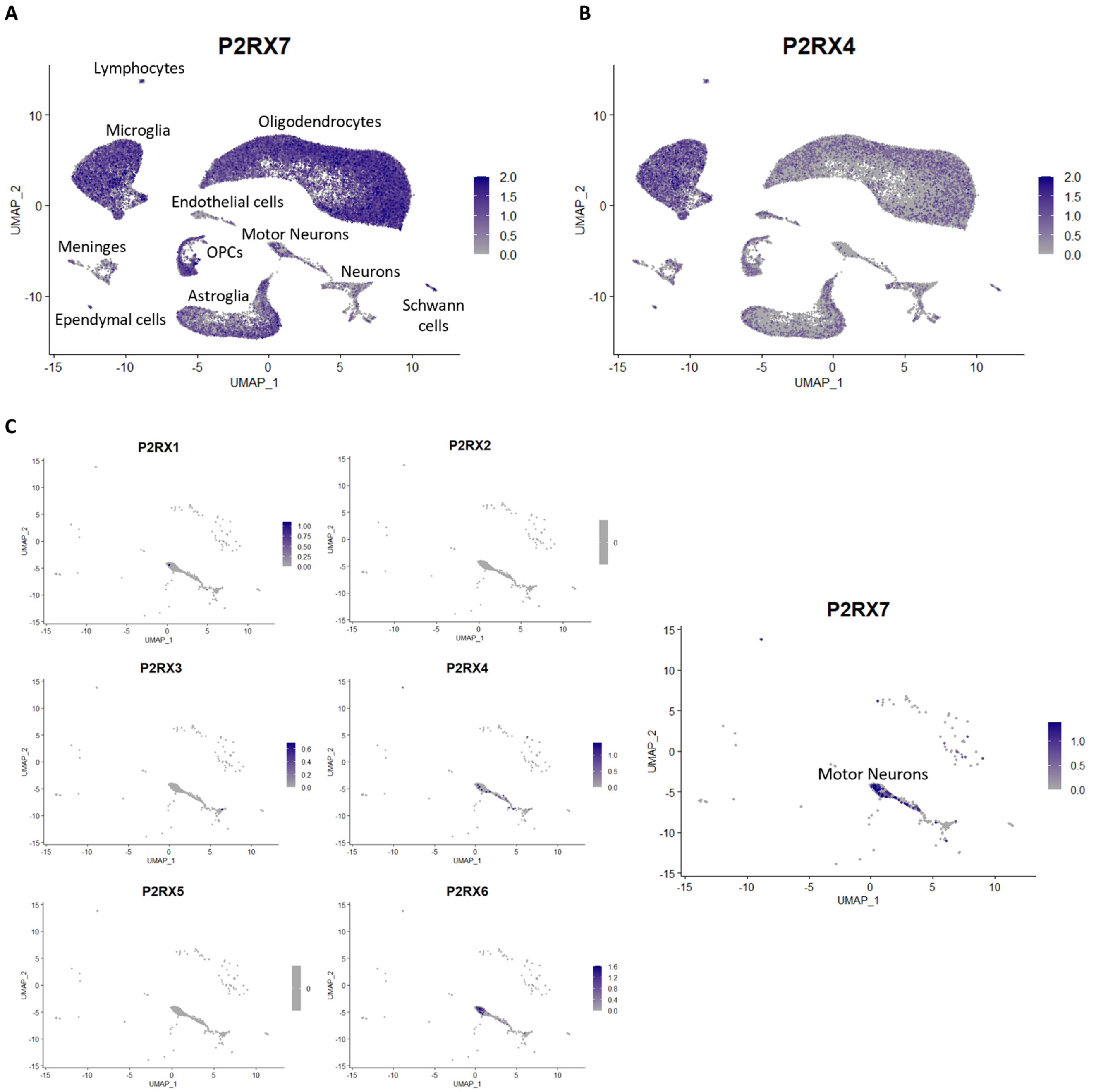

Supplementary 4

A

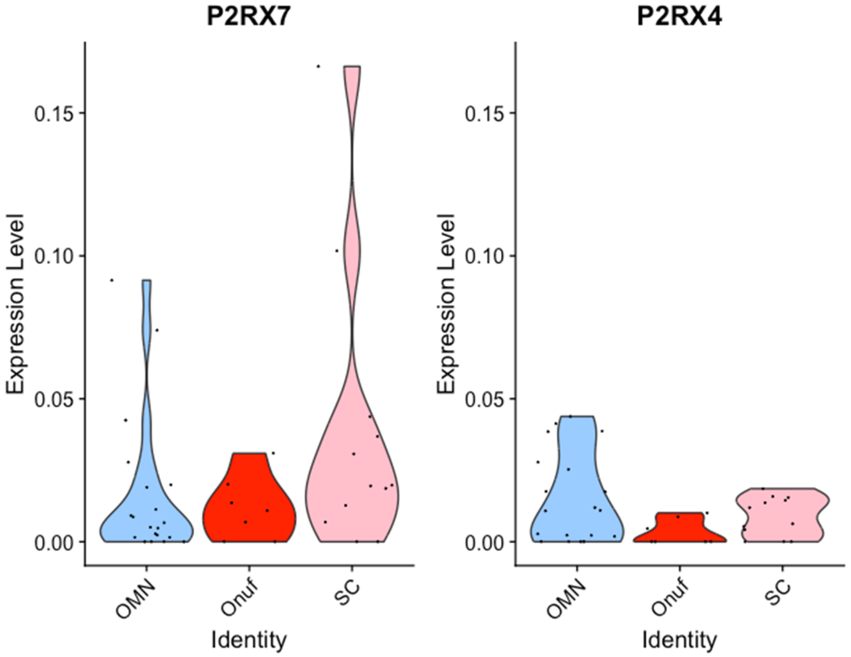

B

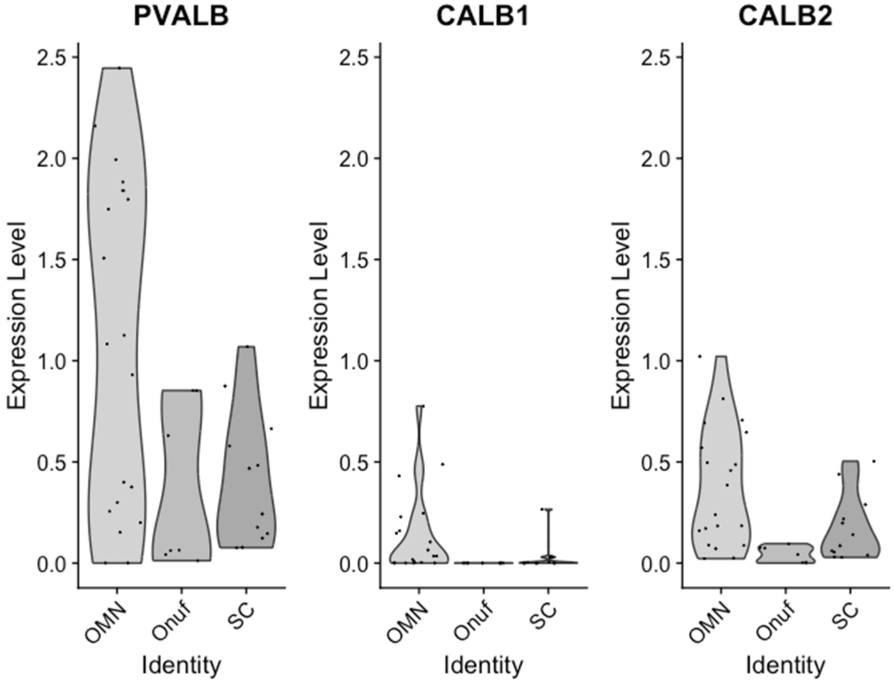

Supplementary 5

A

Spinal hiPSC-MN differentiation protocol

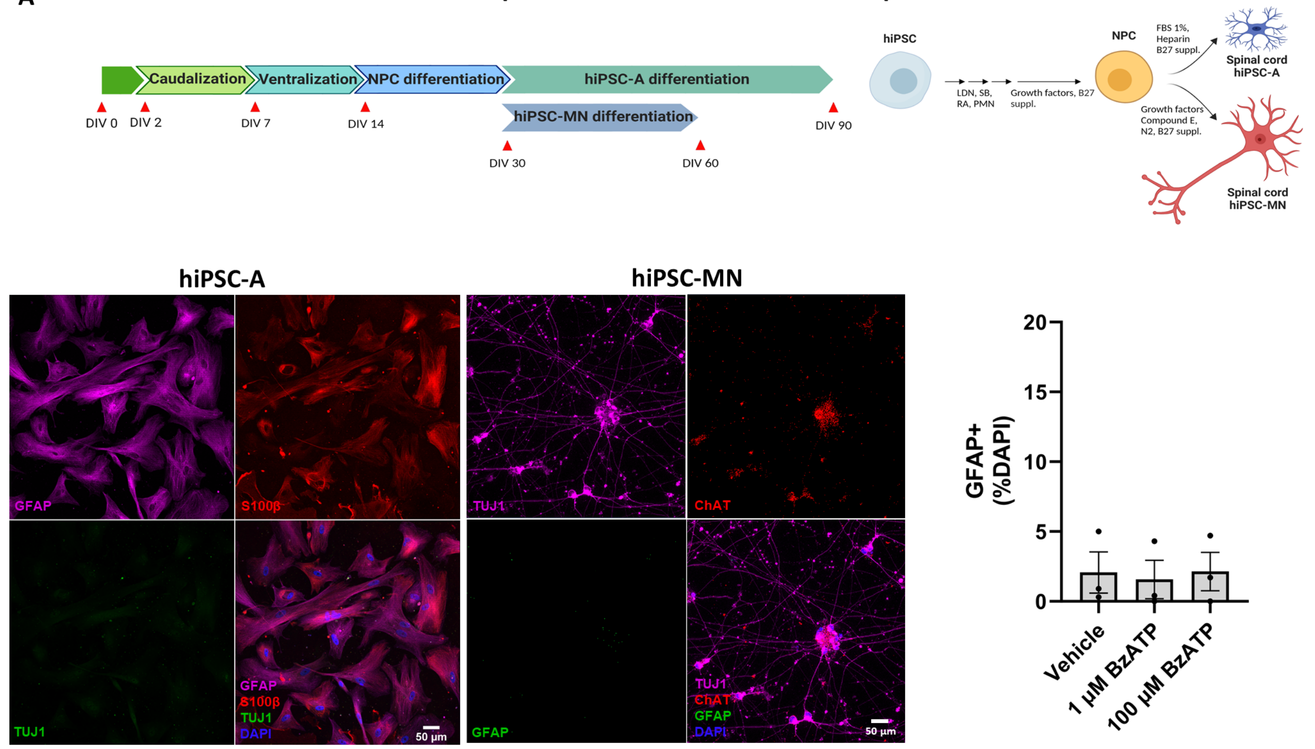

B

Cortical hiPSC-MN differentiation protocol

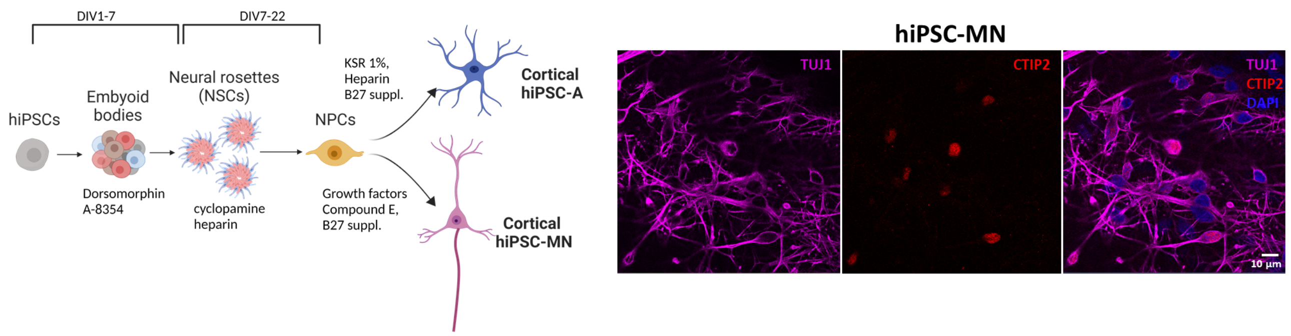

Supplementary 6

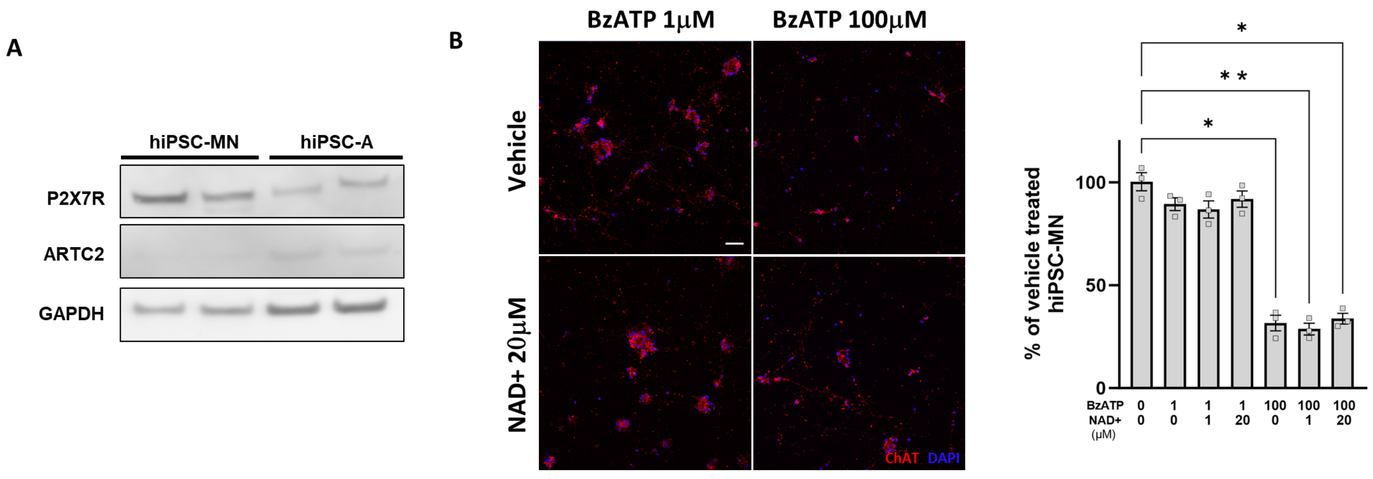

Supplement: szae074_suppl_Supplementary_Figures_S1-S6 [file szae074_suppl_supplementary_figures_s1-s6.pdf]
